# A preclinical study: correlation between PD-L1 PET imaging and the prediction of therapy efficacy of MC38 tumor with ^68^Ga-labeled PD-L1 targeted nanobody

**DOI:** 10.18632/aging.202981

**Published:** 2021-04-27

**Authors:** Songbing Qin, Yang Yu, Hui Guan, Yanling Yang, Fenghao Sun, Yan Sun, Jiaxing Zhu, Ligang Xing, Jinming Yu, Xiaorong Sun

**Affiliations:** 1 Tianjin Medical University, Tianjin 300070, P.R. China; 2 Department of Radiation Oncology, First Affiliated Hospital of Soochow University, Suzhou 215006, P.R. China; 3 School of Graduate Studies, Shandong First Medical University and Shandong Academy of Medical Sciences, Jinan 271099, P.R. China; 4 Department of Radiation Oncology, Shandong Cancer Hospital and Institute, Shandong First Medical University and Shandong Academy of Medical Sciences, Jinan 250017, P.R. China; 5 Department of Radiation Oncology, The Fourth People’s Hospital of Jinan, Jinan 250031, P.R. China; 6 School of Pharmacy, Yantai University, Yantai 264003, P.R. China; 7 Smart Nuclide Biotech, Suzhou 215123, P.R. China; 8 School of Clinical Medicine, Weifang Medical University, Weifang 261053, P.R. China; 9 Department of Nuclear Medicine, Shandong Cancer Hospital and Institute, Shandong First Medical University and Shandong Academy of Medical Sciences, Jinan 250017, P.R. China

**Keywords:** positron emission tomography (PET), immunotherapy, programmed death-ligand 1 (PD-L1), MC38 tumor

## Abstract

Although immunotherapy has achieved great clinical success in clinical outcomes, especially the anti-PD-1 or anti-PD-L1 antibodies, not all patients respond to anti-PD-1 immunotherapy. It is urgently required for a clinical diagnosis to develop non-invasive imaging meditated strategy for assessing the expression level of PD-L1 in tumors. In this work, a ^68^Ga-labeled single-domain antibody tracer, ^68^Ga-NOTA-Nb109, was designed for specific and noninvasive imaging of PD-L1 expression in an MC38 tumor-bearing mouse model. Comprehensive studies including Positron Emission Tomography (PET), biodistribution, blocking studies, immunohistochemistry, and immunotherapy, have been performed in differences PD-L1 expression tumor-bearing models. These results revealed that ^68^Ga-NOTA-Nb109 specifically accumulated in the MC38-hPD-L1 tumor. The content of this nanobody in MC38 hPD-L1 tumor and MC38 Mixed tumor was 8.2 ± 1.3, 7.3 ± 1.2, 3.7 ± 1.5, 2.3 ± 1.2%ID/g and 7.5 ± 1.4, 3.6 ± 1.7, 1.7 ± 0.6, 1.2 ± 0.5%ID/g at 0.5, 1, 1.5, 2 hours post-injection, respectively. ^68^Ga-NOTA-Nb109 has the potential to further noninvasive PET imaging and therapy effectiveness assessments based on the PD-L1 status in tumors. To explore the possible synergistic effects of immunotherapy combined with chemotherapy, MC38 xenografts with different sensitivity to PD-L1 blockade were established. In addition, we found that PD-1 blockade also had efficacy on the PD-L1 knockout tumors. RT-PCR and immunofluorescence analysis were used to detect the expression of PD-L1. It was observed that both mouse and human PD-L1 expressed among three types of MC38 tumors. These results suggest that PD-L1 on tumor cells affect the efficacy, but it on host myeloid cells might be essential for checkpoint blockade. Moreover, anti–PD-1 treatment activates tumor-reactive CD103^+^ CD39^+^ CD8+T cells (TILs) in tumor microenvironment.

## INTRODUCTION

The programmed cell death protein-1 (PD-1)/programmed death receptor ligand 1 (PD-L1) axis, which plays a critical role in helping cancer cells to escape from the immune response, has been extensively studied in cancer immunotherapy [1]. When the PD-1 receptor binds to its ligands PD-L1, the activation of T cells is inhibited. Although PD-1/PD-L1 checkpoint block therapy has changed the management of various tumors and obtained unexpected response [2, 3], lots of cancer patients failed in this new treatment [4]. Some studies have shown that the tumors PD-L1 protein levels can predict the efficacy of PD-1/PD-L1 checkpoint block therapy [5–7]. So it is crucial to select patients who can benefit from immune checkpoint blockades therapy by analyzing the PD-L1 expression in patients before treatment.

Currently, the status of PD-L1 is determined by immunohistochemistry (IHC) method. However, checkpoint molecules are highly dynamic, and heterogeneity and the change of their expression are difficult to obtain, as single time-point biopsies provide limited information throughout a treatment regimen [8]. Recently, non-invasive techniques, such as molecular imaging, can provide real-time data of the total PD-L1 expression in all lesions, complementing the existing immunohistochemical methods [9]. Some studies have shown the advantage of positron emission tomography (PET) imaging with radiolabeled anti-PD-L1 antibodies on the detection of PD-L1 expression over IHC [10]. Meanwhile, some clinical studies have confirmed the potential of PD-L1 PET imaging in cancer patients by the correlations between prognosis and tracer accumulation levels [11, 12]. PD-L1 PET imaging can present the summary amount of PD-L1 expression in cancer patients, both in primary tumors and metastases. Thus, visualizing this expression and the change in different therapies could not only provide scientific insights into synergistic mechanisms but may also benefit cancer patients in making more rational treatment decisions.

Nanobodies of high affinity and specificity can be used as an efficient PET imaging agent of PD-L1, which could increase the uptake in PD-L1-positive tumors and lower the background signal [13]. In this work, Nb109, a non-blocking nanobody with a high selective affinity for PD-L1, was radiolabeled with ^68^Ga (t_1/2_=49.79min) and it showed high potential applications for guiding immunotherapy. As our recent study [14], the biodistribution and PET imaging studies of ^68^Ga-NOTA-Nb109 demonstrate its PD-L1 specificity in tumor models with PD-L1 expression variation. In addition, such non-invasive real-time imaging tracer can distinguish the expression level of PD-L1 in malignant tumors. For further clinical conversion, we identified the ability of ^68^Ga-NOTA-Nb109 in PD-L1 imaging expression in MC38 xenograft tumors and explored whether it can assess the effectiveness of treatment.

Besides, the resistance and relapse of the tumor often require immunotherapy by combining other therapies [15]. Even so, its mechanism and the involved dynamic process are still mostly a mystery. Sindilizumab, a new PD-1 targeting antibody, could effectively inhibit tumor growth with higher PD-L1 expression but not the lower one. This antibody is a human immunoglobulin G4 (IgG4) monoclonal antibody (HuMAb), which can bind to the PD-1 receptor, blocking the interaction with PD-L1 and PD-L2, and prevent the tumor immunosuppressive response mediated by PD-1 pathway. The binding sites are different between ^68^Ga-NOTA-Nb109 and Sindilizumab to receptor, so there is theoretically no impact on the specific binding of the probe to PD-L1. Then we used Sindilizumab on the xenograft models and want to explore the possibility of molecular imaging in predicting curative effect for the combined treatment.

## MATERIALS AND METHODS

### Materials

All solvents and reagents were provided by Sigma-Aldrich (Beijing, China). p-SCN-Bn-NOTA was from Macrocyclics (Dallas, USA). The PD-1 anti-body Sintilimab was kindly provided by Innovent Co. Ltd (Suzhou, China). The Nb109 antibody was obtained from Smart Nuclide Biotech (Suzhou, China). The mass spectra were measured by the high-resolution LTQ-Orbitrap XL mass spectrometer connected to a heated electrospray ionization source (Thermo Scientific, USA). The data were processed by Thermo Biopharma Finder 3.0. 68Ga was obtained from a 68Ga/68Ge generator (IGG-100, Eckert and Ziegler, Germany). High-Performance Liquid Chromatography (HPLC) was performed on Waters 2998 with a size-exclusion chromatogram (SEC) (G3000SWXL, TOSOH, Japan).

### Antibody conjugation

The Nb109 antibody was purified by size-exclusion chromatography (SEC) HPLC, in which 0.01 M sodium phosphate buffer (pH 7.4) was using as the mobile phase at the flow rate of 1 mL/min and concentrated by ultrafiltration centrifugal tube (Amicon^®^ Ultra-2 30 kDa centrifugal filter). Then the pH of the resulting solution was adjusted to 8.5-9.0 with PBS buffer (pH = 9). P-SCN-Bn-NOTA dissolved in DMSO was added in the solution. After incubating at 37° C for 1 hour, the antibody conjugate was purified twice with ultrafiltration centrifugal tube and PBS (pH=7.4). The antibody complex (NOTA-Nb109) stock solution was stored at 4° C.

### Synthesis of the probe 68Ga-NOTA-Nb109

Add the conjugated complex (NOTA-Nb109) solution into the tube. And the reaction system pH was adjusted to 4.0 by adding 0.05 M HCl, following adding an aliquot of 68Ga ^3+^ (1 mL) in sodium acetate (225 μL of 0.25 M). After incubating for 10 min at 37° C, the antibody conjugate purifying was performed through PD-10 column. 68Ga-NOTA-Nb109 was eluted with saline, following with quality control for further study.

### Cell lines and culture conditions

The colon cancer cell line MC38 was used. The MC38 stably expressing human PD-L1 cell line (MC38-hPD-L1) and MC38 PD-L1 knockout (MC38-KO) cell line were kindly provided by Smart Nuclide Biotech (Suzhou, China). Cell lines were cultured in RPMI 1640 with 10% fetal bovine serum (Gibco), with antibiotics (penicillin 100 IU/mL, and streptomycin 100 mg/mL). All the cells were cultured in an incubator at 37° C with 5% CO_2_ atmosphere.

### Flow cytometry detective for PD-L1 expression

The expression of PD-L1 on three different types of cells, which include MC38-hPD-L1, MC38 wild type and MC38-KO was analyzed using PD-L1 antibody (Clone 44716, ab252436) by Beckman Coulter Cytomics FC 500 MPL (USA) according to procedures described previously [14].

### Animal studies

Female human PD-1 transgenic C57BL/c mice (18-22 g; 5-7 weeks old) were obtained from Model Organisms Co., Ltd (Shanghai, China). Mice were inoculated subcutaneously with 1×10^6^ different MC38 cells. To further demonstrate the PD-L1 targeting ability to ^68^Ga-NOTA-Nb109 and eliminate the individual differences, in each mouse, three different cancer cells, MC38-hPD-L1, the mixture of MC38-hPD-L1/MC38-KO (1/1, v/v) and MC38-KO were inoculated at the left hind leg, right hind-leg and right fore-leg respectively. Mice were used for imaging or biodistribution experiments when tumors reached about 250~350 mm^3^. All experiments for animal research were conducted according to the principles established by the ethical committee of Shandong Cancer Hospital.

### *Ex vivo* biodistribution

Mice with three tumors xenografts models were randomly divided into two groups (n=5) and both groups were received an intravenous injection of 3.7 MBq of ^68^Ga-NOTA-Nb109. Biodistribution studies were performed as described previously [14]. After injection at 1 h and 2 hs, the mice were sacrificed, and the main organs were collected, weighed, and counted by a g counter. The uptake of the radiotracer was expressed as percentage injected dose per gram of tissue (%ID/g), which were measured in triplicate. Biodistribution data was expressed as mean ± the standard error of the mean (SEM).

### MicroPET imaging studies

MicroPET imaging was performed on an Inveon microPET scanner (SiemensMedical Solutions, Germany). Xenograft mice were injected intravenously with 4.0-5.0 MBq ^68^Ga-NOTA-Nb109 and imaged (10 min) at 0.5, 1, 2, 4 hours post-injection. The mice were pre-treated with Sindilizumab (5 mg/kg) one day in advance in the blocking group. The mice were anesthetized with 1.5%–2% isoflurane in 0.5 L/min flow of oxygen. Dynamic images had been collected continuously in two hours. All the images were reconstructed using three-dimensional ordered subset expectation maximization (OSEM 3D/SP-MAP) without attenuation correction and then processed through the Siemens Inveon Research Workplace (IRW2.0.0.1050). The interest regions were drawn over both tumors and central organs, and the average signal levels in the regions were measured.

### Tumor treatment models and growth delay assays

**Figure 1:**
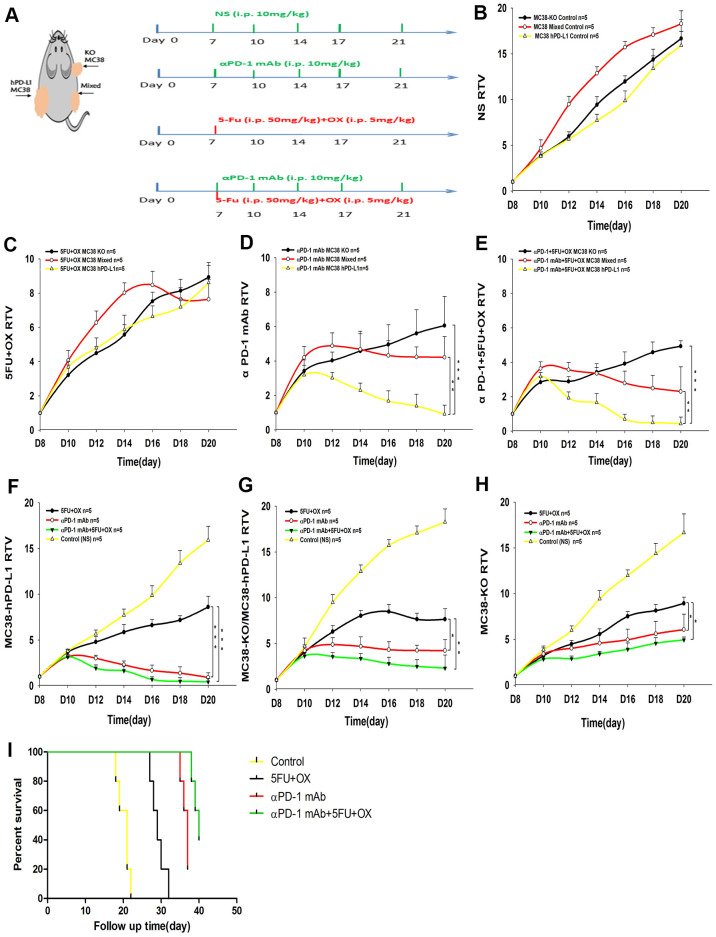
**Effect of different treatments on tumor growth in tumor-bearing mice.** (**A**) Treatment schedules. (**B**–**H**) Tumor growth delay cure (n=5) (**B**) The growth cure of different tumors with normal saline. (MC38-KO, black; MC38 Mixed, red; MC38 hPD-L1, yellow). (**C**–**E**) Different treatment methods for different tumors. (**F**–**H**) The growth of different tumors treated with 5Fu and Oxaliplatin (OX), anti-PD-1, or combined treatment. (**I**) Mice survival analysis. ^***^P <0.001, ^**^P <0.01, ^*^P <0.05.

Therapy commenced when tumor volumes reached 60-80 mm^3^ on 7-8 days after cell inoculation (as day 0). Then mice were randomly divided into the following four groups (n = 5, per group): i) Untreated control, mice were injected with 100 μL of 0.9% sodium chloride;ii) anti PD-1 alone, mice were administered with Sindilizumab 10 mg/kg every other day for a total of five treatments; iii) chemotherapy alone, mice were intravenously injected with 5-Fu (50 mg/kg) and Oxa (Oxaliplatin; 5mg/kg) at the first day for once; iv) and chemotherapy plus anti-PD-1 therapy, mice were given with Sindilizumab, 5-Fu, and Oxa as the above methods and dosage. The different treatment therapies are shown in Figure 1A. The tumors were measured with a digital caliper (volume=length×width×width×0.5) [16]. As described in our previous study [17], the relative tumor volume (RTV) was calculated as follows: RTV = Vt / V_0_, where Vt is the volume at any given time and V_0_ is the initial volume before treatment. The RTV values were recorded every two days, and the tumor growth delay curve was analyzed. During animal experiments, the survival time was recorded from the date of treatment initiation to the date of death or sacrifice when the tumor volume had reached 2000 mm^3^.

### RT-PCR analysis

Total RNA from different tumors tissues was extracted by Trizol regent and reverse transcription was performed using the Servicebio^®^RT First Strand cDNA Synthesis Kit (G3330, Servicebio, Wuhan, China). Real-time PCR was performed using the 2×SYBRGreen qPCR Master Mix (G3322, Servicebio). Primers used in this experiment were as follows: Mouse PD-L1 forward: 5'-CTACGGTGGTGCGGACTACAA-3', reverse: 5'-GGATAACCCTCGGCCTGACATA-3'; Human PD-L1 forward:5'- GCCGAAGTCATCTGGACAAGC-3', reverse:5'- GTGTTGATTCTCAGTGTGCTGGTCA-3'; GAPDH forward:5'- CCTCGTCCCGTAGACAAAATG-3', reverse:5'- TGAGGTCAATGAAGGGGTCGT-3'. The 2-ΔΔCt method was used to analyse the relative gene expression.

### IHC and IF staining

Following sacrifice of the mice, half part of the tumors were embedded in optimal cutting medium (OCT 4583; Sakura Finetek), and frozen in -80 degree. The rest part of the tumors were fixed in 10% formalin overnight (within 12 h) at 25° C and embedded in paraffin blocks. The 4-μm thick sections were prepared for IHC and dewaxed.

After antigen retrieval using 10 mmol/l citrate buffer, sections were incubated with 3% H_2_O_2_ and blocked with 5% BSA for 1 h. Then the sections were added with the primary anti-PD-L1 antibody at 4° C overnight. After rewarming to room temperature, the sections were incubated with secondary antibodies using the two-step polymer HRP detection system (OriGene Technologies, Inc.). The slices were visualized with 3,3-diaminobenzidine and then counterstained with haematoxylin.

For IF, 8-10 μm sections were fixed with 4% paraformaldehyde for 20 min and then blocked with 5% BSA at 37° C for 0.5 h. Then the sections were incubated with anti-PD-L1 (ab20592, ab213480, Abcam), CD8, (ab263946, Abcam), CD103(AF5155, Affinity), CD39(DF4031, Affinity) and Caspase-3 (ab13847, Abcam) primary antibodies at 4° C overnight. After being rewarmed for 1 h, samples were washed carefully and incubated with specific secondary antibodies (1:200; Thermo Fisher Scientific, Inc.) for 1 h at 37° C, followed with DAPI for 2min. Fluorescence images were measured using a Nikon H600L ECLIPSE 90i fluorescence microscope (Nikon Corporation; magnification, x200), and IHC images were captured with a light microscope (Olympus Corporation; magnification, x200). CD8 was imaged through red filters. PD-L1 was imaged through red and gray filters. CD39, CD103 and Caspase-3 were imaged by green filters.

### Statistical analysis

The Statistical Package of Social Sciences (version 16.0; SPSS, Chicago, IL, USA) was used for data analyses. Results were shown as the mean ± standard error. We used GraphPad Prism software (version 7.04) and ImageJ software (version 1.8.0) for statistical analyses. P<0.05 was considered statistically significant. (*P < 0.05, **P < 0.01, ***P < 0.001).

## RESULTS

### Characterization and radiochemistry

**Figure 2:**
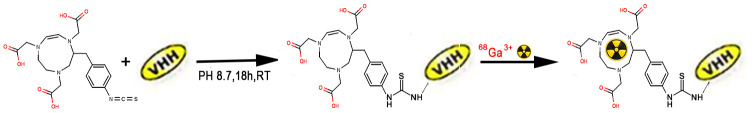
**Chemical structure of ^68^Ga-NOTA-Nb109.** RT, room temperature. VHH, stands for Nb109.

The conjugation scheme and structure of the ^68^Ga-NOTA-Nb109 are shown in Figure 2. As our previous study proved [14], the radiochemical yield, as well as the radiochemical purity of ^68^Ga-NOTA-Nb109, was respectively more than 95% and 98%. The specific activity was calculated to be 25.17 ± 3.26 GBq/μM. In addition, our previous study showed that ^68^Ga-NOTA-Nb109 had excellent stability *in vitro* over 4 h at 75° C.

### Flow cytometry analysis

The expression of PD-L1 in different cell lines was measured by flow cytometry (Supplementary Figures 1, 2).

### PET imaging

**Figure 3:**
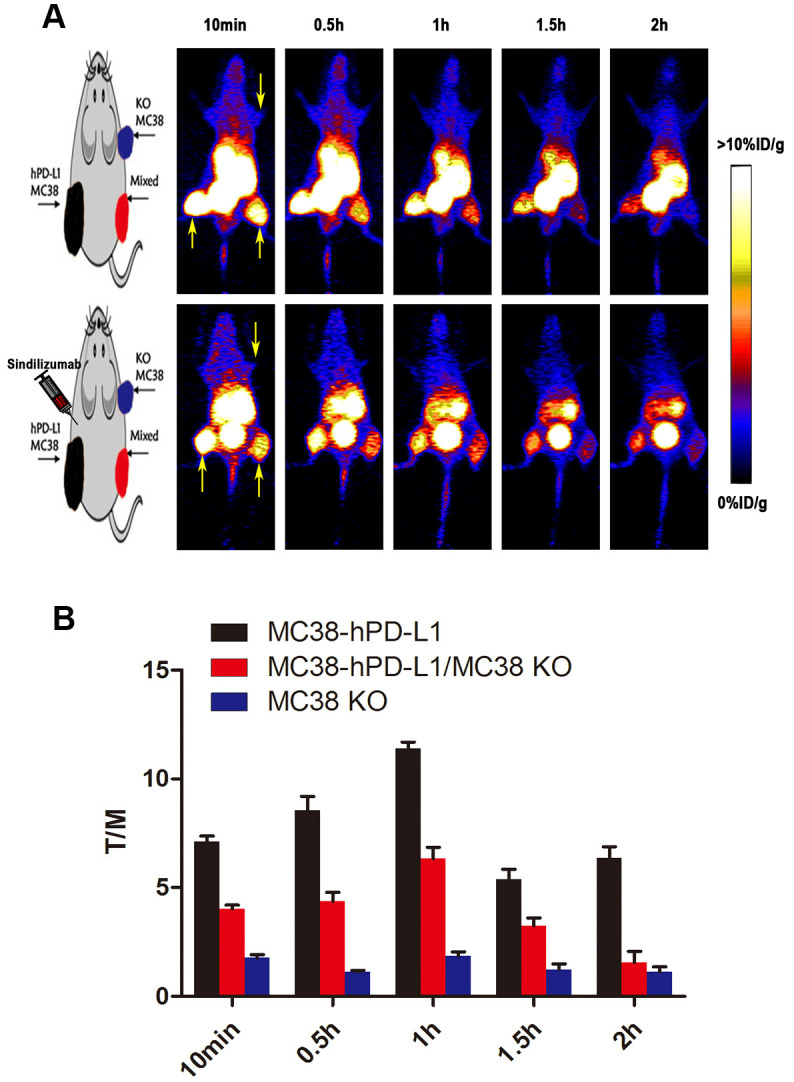
**PET imaging studies of ^68^Ga-NOTA-Nb109.** (**A**) Dynamic PET scanning of MC38 tumor–bearing models (with or without injection of Sindilizumab) over 0–2 h after injection of 4.0–5.0 MBq of ^68^Ga-NOTA-Nb109. (n=3, tumors indicated by the yellow arrow). (**B**) Tumor-to-muscle (T/M) ratio of ^68^Ga-NOTA-Nb109 was analyzed according to the quantification analysis of PET images.

Encouraged by the excellent property of ^68^Ga-NOTA-Nb109, its effects *in vivo* were examined in the following imaging. Dynamic PET/CT imaging of ^68^Ga-NOTA-Nb109 at 10 min, 0.5, 1, 1.5, 2 hours after injection was observed. As shown in Figure 3A, after 10 min post-injection, ^68^Ga-NOTA-Nb109 could notably accumulate at tumor sites in mice. The content in MC38 hPD-L1 tumor and MC38 Mixed tumor was 8.2 ± 1.3, 7.3 ± 1.2, 3.7 ± 1.5, 2.3 ± 1.2%ID/g and 7.5 ± 1.4, 3.6 ± 1.7, 1.7 ± 0.6, 1.2 ± 0.5%ID/g at 0.5, 1, 1.5, 2 hours post-injection, respectively. However, MC38 KO tumors were not visible during the entire PET collection process. The images were optimal at 1 h after injection, with the highest uptake ratio of tumor-to-muscle being 11.4±0.29 (In Figure 3B), In tumor–bearing mice pretreated with Sindilizumab (10mg/kg) for 24 h, the tumor uptake of ^68^Ga-NOTA-Nb109 was still observed, proving that Anti-PD1 treatment did not affect the specific binding of ^68^Ga-NOTA-Nb109 with PD-L1.

**Figure 4:**
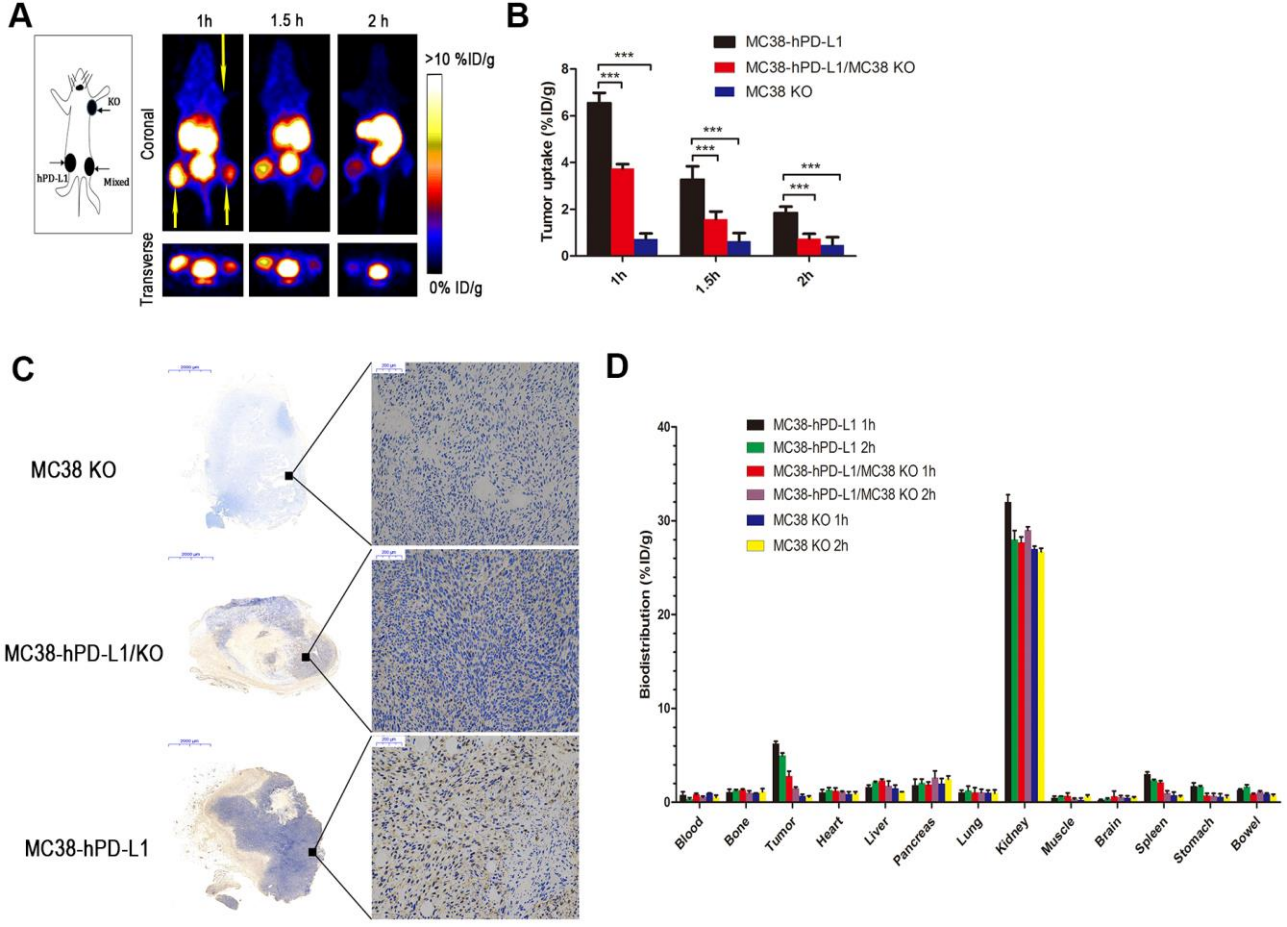
**PET imaging of tumor-bearing models.** (**A**) Static images at 1, 1.5, and 2h after injection of 2.5 MBq of ^68^Ga-NOTA-Nb109 (n=3, tumors indicated by the yellow arrow). (**B**) The uptake of ^68^Ga-NOTA-Nb109 in tumors according to quantification analysis of PET images. (**C**) PD-L1 immunohistochemical staining of tumors (n=5, scale bar =2 mm in the left column and 200 μm in right column). ^***^P <0.001. (**D**) Biodistribution of ^68^Ga-NOTA-Nb109 in major organs at 1 and 2 h (n=5).

PET static scanning was performed on three types of tumor-bearing mice in Figure 4A. As expected, the MC38-hPD-L1 tumor was observed and showed the highest uptake of radioactivity at 1 h (6.56 ± 0.42 %ID/g) (Figure 4A, 4B). For the MC38-hPD-L1/MC38-KO tumor mixture, the content of uptake at 1 h was 3.74 ± 0.19 %ID/g, and it was almost half of that in MC38-hPD-L1 tumor at all time points. For PD-L1-negative MC38-KO tumor, it was not visible during the PET acquisition. The results correlated with PD-L1 expression as determined by IHC in Figure 4C.

### Biodistribution studies

The biodistribution of ^68^Ga-NOTA-Nb109 in tumor–bearing mice at 1 and 2 hours post-injection was shown in Figure 4D. At 1 h post-injection, the kidneys showed relatively high uptake (32.5±1.77 % ID/g), whereas the spleen (3.02±0.24 % ID/g), the pancreas (1.80±0.65 % ID/g), the stomach (1.71±0.36 % ID/g) and remaining organs (<2.0 % ID/g) displayed low uptake. Furthermore, the uptake in MC38-hPD-L1 tumor was rapid and high (6.24± 0.26 % ID/g), and the content of uptake in MC38-hPD-L1/KO tumor was 2.81± 0.52 %ID/g, but that in MC38 KO tumor was only 0.68±0.17 %ID/g. At 2 h after injection, the content in MC38-hPD-L1 tumors and MC38-hPD-L1/KO tumors slightly decreased to 4.97±0.27 and 1.48± 0.16 %ID/g, respectively. In addition, the content in the MC38 KO tumor was only 0.55±0.14 %ID/g.

### Therapy study on xenografts models

**Figure 5:**
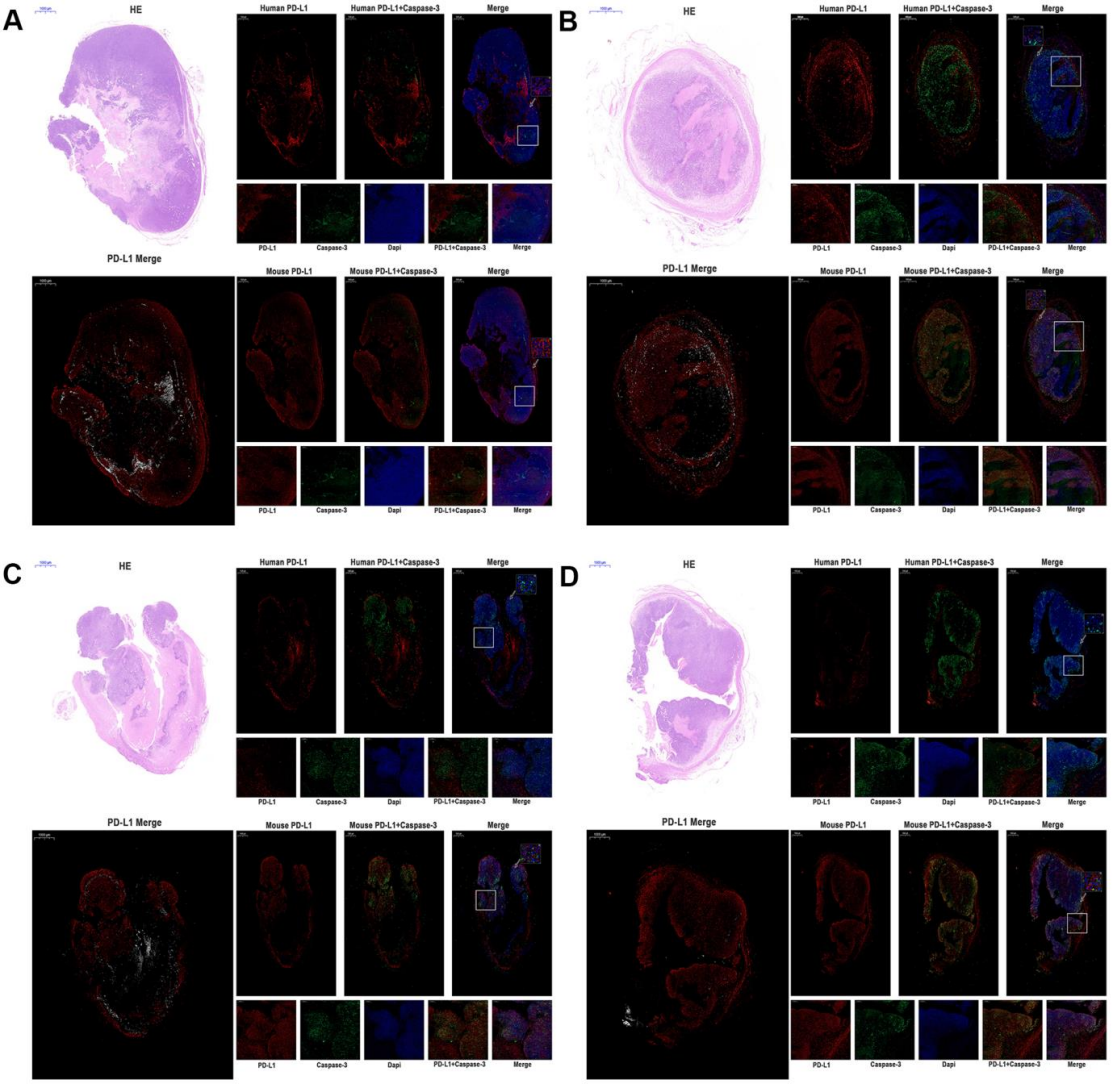
**The expression of human PD-L1, mouse PD-L1, and Caspase-3 were detected by immunofluorescence of MC38 tumors (n=3, Scale bar=1000 μm).** (**A**) MC38-hPD-L1 control (**B**) MC38-hPD-L1 (**C**) MC38-hPD-L1/KO (**D**) MC38-KO Human PD-L1 or Mouse PD-L1 was indicated by red signals; Caspase-3 in tumor or tumor-host was indicated by green signals; nuclei, blue 4’,6-diamidino-2-phenylindole (DAPI) signals. HE staining: upper left. PD-L1 Merge: lower left (Mouse PD-L1, red; Human PD-L1, gray). Local amplifies the area indicated by the white box. Scale bar=200 μm. Inset amplifies the area indicated by the white arrow. Scale bar=50 μm.

For better clinical conversion, we inoculated cancer cells into human PD-1 transgenic mice. Based on numerous ongoing clinical studies for exploring the possible synergy of immunotherapy combined with conventional cancer treatments, we selected the combined therapy, *i.e.*, chemotherapy plus anti-PD-1 therapy (Figure 1A). As shown in Figure 1B, the growth rate of three tumors was no significant difference (MC38-KO, black line; MC38 Mixed, red line; MC38 hPD-L1, yellow line). Next, we compared the therapeutic effects of same treatment method for different tumors. For the tumor growth delay assay in Figure 5C–5E (MC38-KO, black line; MC38 Mixed, red line; MC38 hPD-L1, yellow line), three treatment methods were effective for different tumors. After single-dose 5-Fu and Oxa chemotherapy in Figure 1C, all the three types of tumors showed significant growth delay, but no difference between the tumors. For anti-PD-1 mAb treatment in Figure 1D, all these tumors displayed apparent growth arrest. Importantly, the hPD-L1 group provided the better efficacy than the mixed group and the KO group (MC38 hPD-L1 vs. MC38 mixed group, P<0.01; MC38 hPD-L1 vs. MC38 KO group p<0.001). Similarly results were found in the anti-PD-1 mAb combined with chemotherapy group (Figure 1E, MC38 hPD-L1 vs. MC38 mixed group, P<0.01; MC38 Hpd-L1 vs. MC38-KO, P<0.001). Besides, for the spontaneous tumor regression, the MC38 hPD-L1 group expressed the highest regression rate of 5/14 (35.7%), and the regression rate of MC38-KO group, as well as MC38 mixed group were 2/8 (25%) and 2/8 (25%), respectively.

Then we compared the therapeutic effects of different treatment methods for the same type tumor. The tumor growth delay assay in Figure 1F–1H (Control group, yellow line; Chemotherapy group, black line; anti-PD-1 group, red line; Combined group, green line), for MC38-hPD-L1 tumor, tumor growth was inhibited in both combined treatment group and anti-PD-1 treated alone group, compared with the 5-FU+OX alone group (Figure 1F, Combined vs. Chemotherapy group, P<0.001; anti-PD-1 group vs. Chemotherapy, P<0.001). Similar results were found in both MC38 mixed group (Figure 1G, Combined vs. Chemotherapy group, P<0.01; anti-PD-1 group vs. Chemotherapy, P<0.05) and MC38-KO group (Figure 1H, anti-PD-1 alone vs. Chemotherapy, P<0.05; Combined vs. Chemotherapy group, P<0.05). The survival analysis showed in Figure 1I, it was observed that anti-PD-1 and combined groups had a better survival compared with chemotherapy and control groups. These results showed that, compared with anti-PD-1 alone, the combined therapy did not notably improve the antitumor effect in different tumors and PD1 blockade therapy was effective for MC38-KO groups.

### Both mouse and human PD-L1 expressed in tumors

In the current study, the antitumor effect was different from our expectation, especially the immunotherapy was also effective for the MC38 KO group. Then, we performed qRT-PCR to examine the expression of mouse PD-L1 and human PD-L1 mRNA in four type MC38 tumor issues (MC38-hPD-L1 control, MC38-hPD-L1, MC38-hPD-L1/KO, and MC38-KO). As shown in Supplementary Figure 3A, mouse PD-L1 mRNA was expressed in all MC38 tumor issues and there was no difference between groups. While, results showed in Supplementary Figure 3B, the expression of human PD-L1 mRNA was significantly higher in MC38-hPD-L1 and MC38-hPD-L1/KO groups than that in MC38-KO groups. Thus, these data indicated that both mouse and human PD-L1 expressed in MC38 tumors.

### PD-L1 on host cells might be essential for PD-1 blockade-mediated tumor regression and PD-L1 on tumor cells affect the efficacy

**Figure 6:**
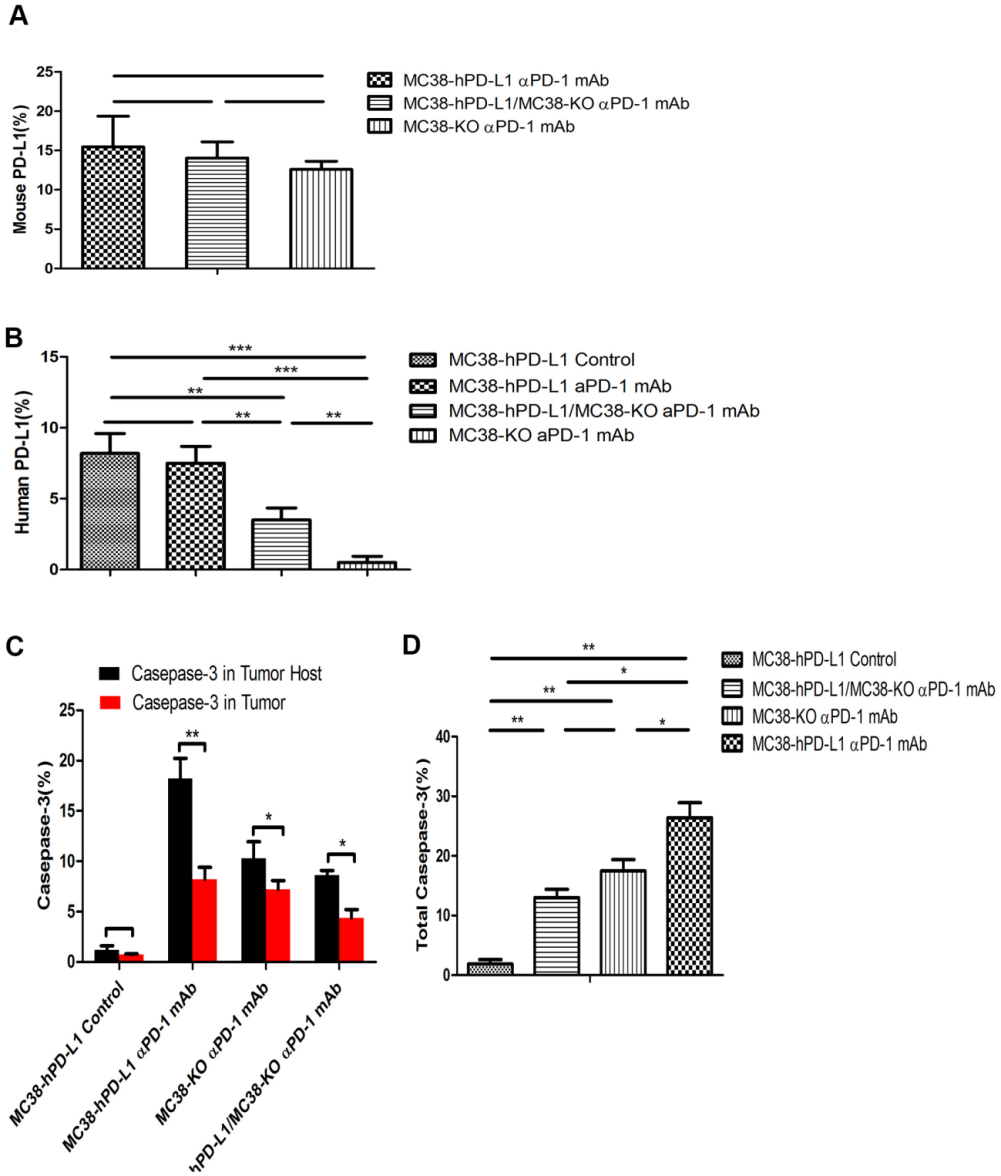
**Fluorescence quantitative analysis.** (**A**) Percentage of mouse PD-L1(PD-L1 in the host) among the different tumors. (**B**) Percentage of human PD-L1(PD-L1 in Tumor) among the different tumors. (**C**) Percentage of caspase-3 in tumor or tumor-host among the different tumors. (**D**) Percentage of total caspase-3 among the different tumors. ^***^P <0.001, ^**^P <0.01, ^*^P <0.05.

Several studies [18, 19] reported that the expression of PD-L1 on host determines tumor treatment efficacy mediated by the PD-L1 pathway blockade. To investigate the function and distribution of PD-L1 in tumors, the IF experiments were performed in the anti-PD-1 alone groups (MC38-hPD-L1 control, MC38-hPD-L1, MC38-hPD-L1/KO, and MC38-KO). In Figure 5A–5D and Figure 6A, 6B, results showed that human PD-L1 (hPD-L1) was clearly expressed near the necrosis area, mainly in membrane and cytoplasm and mouse PD-L1 (mPD-L1) was extensive and relatively uniform, which was expressed primarily on membrane and cytoplasm. The spatial distribution of hPD-L1 and mPD-L1 was complementary. Quantitative analysis showed that mouse PD-L1 was expressed in all MC38 tumor issues and there was no difference between groups (Figure 6A). While, human PD-L1 was significantly higher in MC38-hPD-L1 and MC38-hPD-L1/KO groups than that in MC38-KO groups (MC38 hPD-L1 vs. Control group, P>0.05; MC38 hPD-L1 vs. MC38 KO group, P<0.001; MC38 hPD-L1 vs. MC38 Mixed group, P<0.01; MC38 Mixed vs. Control group, P<0.01; MC38 Mixed vs. MC38 KO group, P>0.05; MC38 KO vs. Control group, P<0.01,Figure 6B). These results were consistent with the results in qRT-PCR.

As the expression of PD-L1 determined the process of antigen extraction [17], we analyzed the caspase-3 induced by human PD-L1 and mouse PD-L1. In Figure 5A–5D, compared with the control groups, caspase-3 expressed widely and distributed in mPD-L1 expression area. The quantitative analysis were showed in Figure 6C, 6D. Caspase-3 in tumor-host was higher than that in tumor (for MC38 hPD-L1 group, P<0.01; for MC38 KO group, P<0.05; for MC38 Mixed group, P<0.05). The total Caspase-3 also had variant between different tumors, which were consistent with the results *in vitro* (MC38 hPD-L1 vs. Control group, P<0.01; MC38 hPD-L1 vs. MC38 KO group, P<0.05; MC38 hPD-L1 vs. MC38 Mixed group, P<0.05; MC38 Mixed vs. Control group, P<0.01; MC38 Mixed vs. MC38 KO group, P>0.05; MC38 KO vs. Control group, P<0.01). Together, these findings demonstrated that although MC38 KO tumor cell did not express hPD-L1 nor mPD-L1 *per se*, the host cell in the xenograft did expressed mPD-L1 after the MC38 KO xenograft was established. The growth of MC38 KO tumor was significantly retarded after given anti-PD-1 treatment as same as the MC38 hPD-L1 tumor and MC38 mixed tumor. We further verified that there was no significant difference in mPD-L1 expression among three types of MC38 tumor based on our RT-RCR data. While caspase-3 induced by PD-L1 had differences between different tumors. Therefore, we inferred that PD-L1 on tumor cells could affects the efficacy to checkpoint blockade, but PD-L1 in host cells might be essential for this response.

### Anti–PD-1 treatment activates tumor-reactive CD8^+^T cells in tumor microenvironment

**Figure 7:**
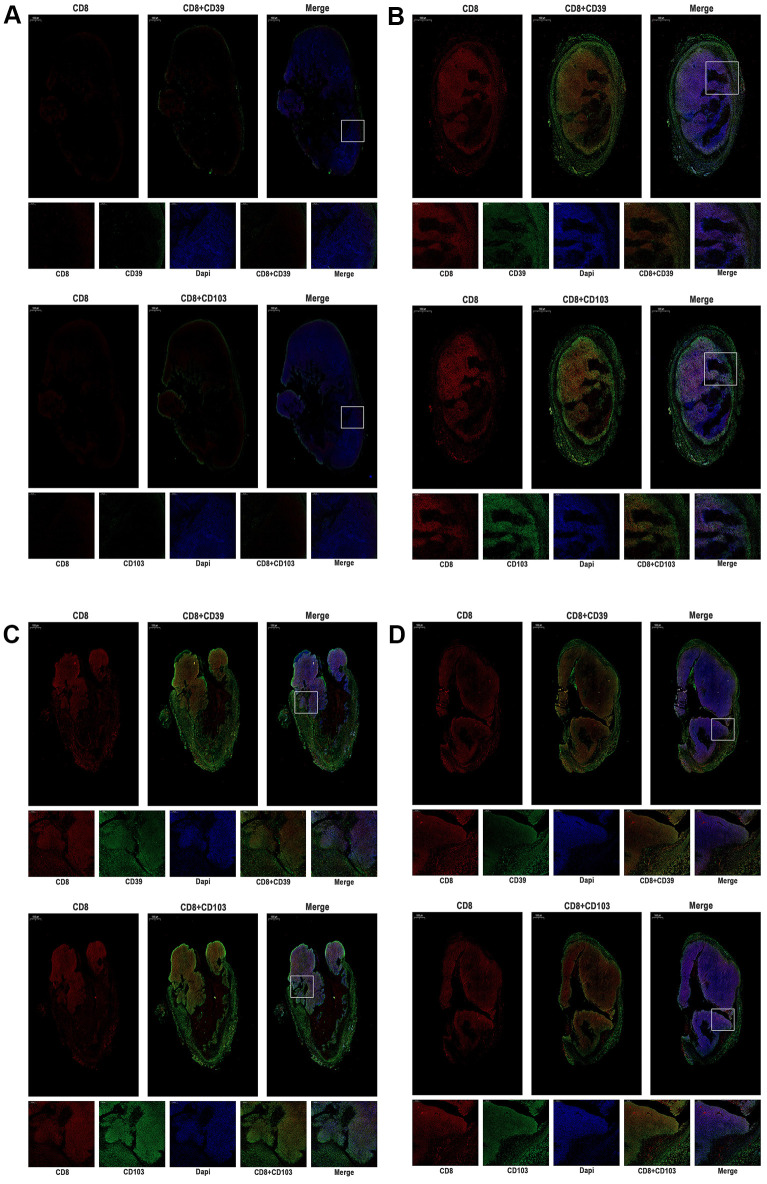
**The expression of CD39, CD103, and CD8 were tested by immunofluorescence of MC38 tumors (n=3, Scale bar=1000 μm).** (**A**) MC38-hPD-L1 control (**B**) MC38-hPD-L1 (**C**) MC38-hPD-L1/KO (**D**) MC38-KO CD8 was indicated by red signals; CD39 or CD103 was indicated by green signals; nuclei, blue 4’,6-diamidino-2-phenylindole (DAPI) signals. Local amplifies the area indicated by the white box. Scale bar=200 μm.

Given that anti–PD-1 treatment induced antitumor effects in MC38 tumor-bearing mice, we further evaluated the expression of tumor immunity T cell in the mouse tumor microenvironment. Human and murine studies [20–22] showed that CD103^+^CD39^+^ CD8 tumor-infiltrating immune cells (CD8 TILs) have a high frequency of tumor-reactive cells, which have a distinct TCR repertoire. Besides, tumor-reactive CD8T cells, rather than the non-functional bystander CD8T cells, have the function of recognizing and killing autologous tumor cells. In our study, we further assessed the expression of CD103, CD39, and CD8 in different tumors. In Figure 7A, 7B, compared with the control group, results showed that the expression of intense CD8 was higher in MC38-hPD-L1 tumors. At the same time, the expression of CD103 and CD39 was increased in the anti-PD-1 treatment groups. MC38 Mixed and MC38 KO tumors treated with anti-PD-1 also displayed a higher frequency of CD103^+^CD39^+^ CD8 TILs (Figure 7C, 7D). These data suggested that anti-PD-1 therapy increase the tumor-reactive CD8 T cells, promoting tumor apoptosis.

## DISCUSSION

The PD-1/PD-L1 pathway checkpoint block therapy has shown impressive clinical results. IHC analysis of tumor biopsies to assess immune checkpoint targeted expression is the routine clinical examination method [8]. However, checkpoint molecules are highly dynamic and heterogeneous. Thus, a new method that can better understand the spatiotemporal dynamics of the tumor-immune microenvironment is crucial for developing effective therapies of clinical application [9].

Recently, several preclinical studies of radiolabeled PD-L1 imaging agents have been published [10, 23–27]. Additionally, several clinical trials have been proceeding [28, 29]. These agents showed high specificity for imaging PD-L1 expression levels in a variety of cancers, which might be a potential method to guide clinical tumor immunotherapy. Among them, nanobodies can be used as an ideal PET imaging agent of PD-L1. In clinics, for the limited effect of immunotherapy alone on some PD-L1 positive tumors [4, 8], immunotherapy combined with other strategies is necessary to improve the synergistic effect.

In this study, we used a human PD-1 targeting antibody in murine models transfected human PD-1 gene for PD-L1 mapping and immunotherapy, to explore the possibility of PET imaging probe for evaluating the efficacy. ^68^Ga-NOTA-Nb109, as a PET imaging probe, had shown promising results because of its high purity and stability. PET imaging results showed a higher tumor uptake in MC38-hPD-L1 tumor than that in MC38-hPD-L1/KO tumor mixture and MC38 KO tumor, which demonstrated that ^68^Ga-NOTA-Nb109 could selectively and intensely accumulate at PD-L1 positive tumor. These results were further confirmed by our IHC analysis. From the PET imaging and biodistribution studies, the tracer was mainly retained at kidney sites. The tumor radioactivity remains relatively stable for 2 hours. ^68^Ga-NOTA-Nb109 exhibited promising target-to-background ratios in MC38-hPD-L1 tumor and MC38-hPD-L1/KO tumor (11.4±0.29 and 6.33±0.53 tumor-to-muscle ratios in 1h, respectively). By contrast, due to its low uptake in MC38 KO tumor, the target-to-background ratios were low, and tumors were invisible during the imaging process. In addition, before and after the injection of Sindilizumab, there was also no effect on the tumor uptake of ^68^Ga-NOTA-Nb109, as similar tumor uptake and images were obtained. This finding demonstrated the binding sites were different between ^68^Ga-NOTA-Nb109 and anti-PD-1 antibodies to PD-L1. ^68^Ga-NOTA-Nb109 has a great potential in PD-L1 clinical testing and evaluation of prognosis.

More importantly, the PD-L1 mapping from PET scan displays strong corroborative evidence on the immunotherapy effect of different immunocompetent tumors. In the tumor growth delay study, after one course of the antibody Sindilizumab, MC38-hPD-L1 tumor and MC38-hPD-L1/KO tumor was suppressed, whereas an effect on PD-1 blockade efficacy was also observed on MC38-KO tumor. Furthermore, we evaluated PD-L1 on tumor or host cells in different humanized tumor models, suggesting that PD-L1 on host might be essential for PD-L1 and PD-1 checkpoint block antitumor immunity therapy. Lin et al*.* [18] and Tang et al*.* [19] reported similar results that PD-L1 in host myeloid cells was essential for the response to checkpoint blockade. Compared with these studies, we also found that the expression level of PD-L1 on tumor cells affected the response to checkpoint blockade, which requires further verification on other species tumors. However, to develop the combination with chemotherapy and anti-PD-1 therapy in our study, tumor growth was not further inhibited which may be related to the better immunogenicity of MC38 tumor. We also found that anti–PD-1 therapy activates tumor-reactive CD8^+^T cells in tumor microenvironment.

There are several advantages in our present study. First, we used the newly developed non-blocking nanobody with a high specific affinity for human PD-L1, which bound at non-functional sites and did not affect the specific binding of PD1/ PD-L1 blockade, while previous published studies [5, 10, 23, 24, 30–32] often used anti-human PD-L1 antibodies as radiolabeled PD-L1 imaging agents. Second, we utilized human PD-L1 tumor models and human PD-1 transgenic mice, simulated the interaction between human anti-PD-1 antibody and PD1/PD-L1 target in mice, so that our results could be more easily translated in clinical than other certain studies which use murine systems. Third, compared with other studies that constructed different types of tumors in one mouse, our strategy was to construct the same tumor with different PD-L1 expressions. It can avoid the interference caused by the specificity of different tumors.

The aim of this study is to expand the application of Nb109 and explore the possibility of Nb109 to predict the treatment effect, but there are several limitations should be acknowledged. Compared with the study by Emily BE et al. [33] observed that radiolabeled PD-L1 imaging PET could monitor changes of tumor PD-L1 expression followed radiotherapy. One limitation of our study is that no PET imaging was investigated after different treatments. Second, PD-L1 expression is expected to change in different cell lines and different treatment regimens, then the results of the combined treatment strategy obtained in this work may not generally apply to all types of cancer and treatment models. Third, the results PD-L1 in host cells might be essential for the response to checkpoint blockade need to be further verified by PD-L1-and PD-1- deficient mice. Further studies are expected to clarify the precise mechanism.

## CONCLUSIONS

In conclusion, ^68^GaNOTA-Nb109, a newly developed ^68^Ga-labeled nanobody as a PET probe to assess the PD-L1 expression can monitor changes in PD-L1 expression. Additionally, the therapeutic potency of the new PD-1 targeting antibody, Sindilizumab, was evaluated in tumor-bearing mice and inhibited the PD-L1 positive tumor successfully, particularly the PD-L1 knockout tumor. The host immune system might be essential for PD-L1 and PD-1 blockade therapy, which may mechanistically explain for this potential therapeutic efficacy. Further studies are expected to clarify the precise mechanism and to promote the application of PD-L1 PET.

### Ethics approval

The animal experiments were approved by the Animal Ethics Committee at the Shandong Cancer Hospital Affiliated to Shandong University (Jinan, China).

## Supplementary Material

Supplementary Figures
