# Supplementary material for: A preclinical study: correlation between PD-L1 PET imaging and the prediction of therapy efficacy of MC38 tumor with 68Ga-labeled PD-L1 targeted nanobody
Source: Aging (Albany NY). 2021 Apr 27;13(9):13006–22. doi: 10.18632/aging.202981 (PMC8148448; doi:10.18632/aging.202981)
Supplement: Supplementary Figures [file aging-13-202981-s001.pdf]

SUPPLEMENTARY FIGURES

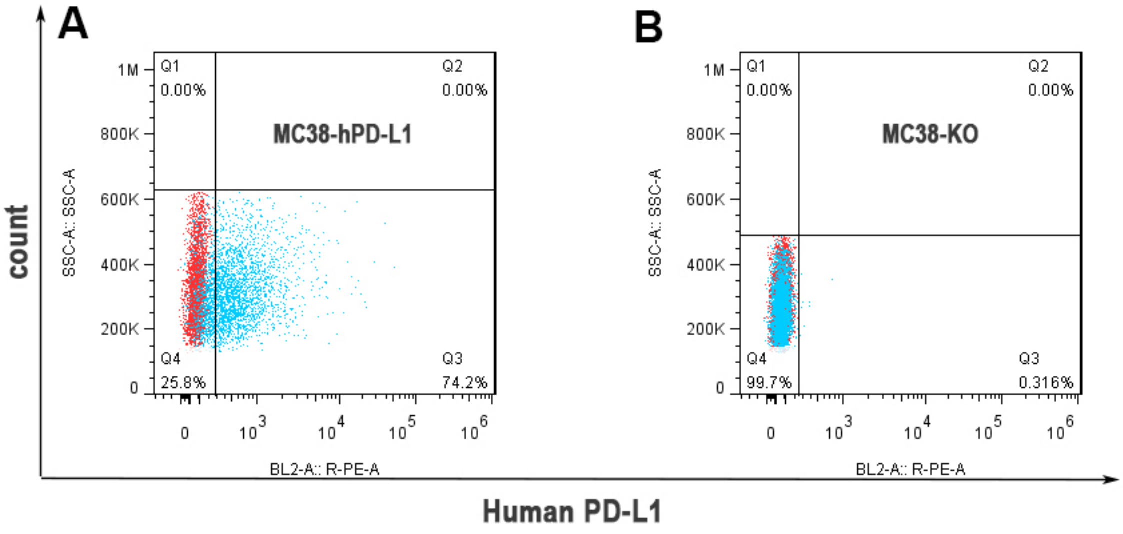

Supplementary Figure 1. The expression of human PD-L1 in MC38-hPD-L1 (**A**) and MC38-KO (**B**) cell lines were measured by flow cytometry.

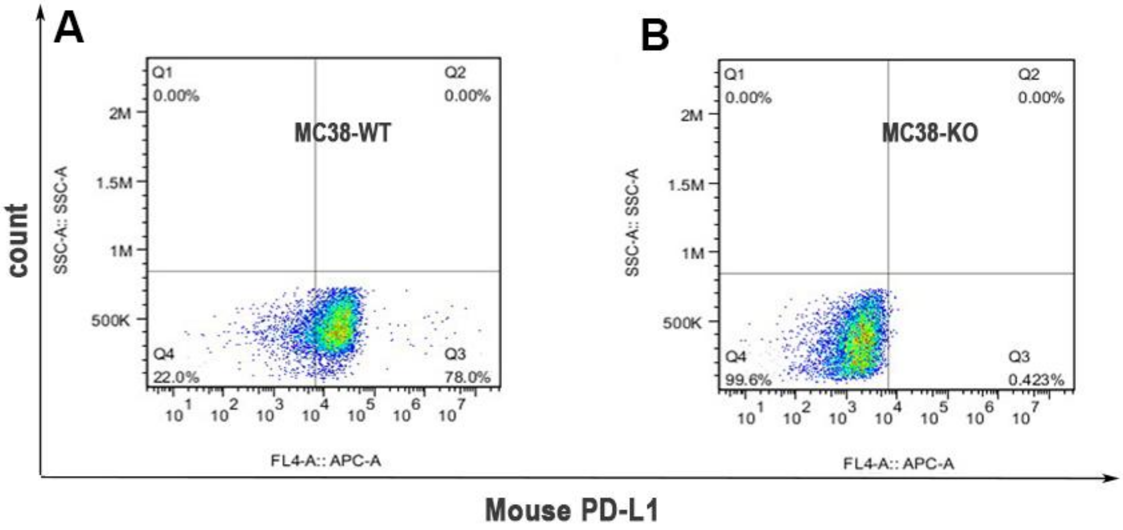

Supplementary Figure 2. The expression of mouse PD-L1 in MC38 wild type (**A**) and MC38-KO (**B**) cell lines were measured by flow cytometry.

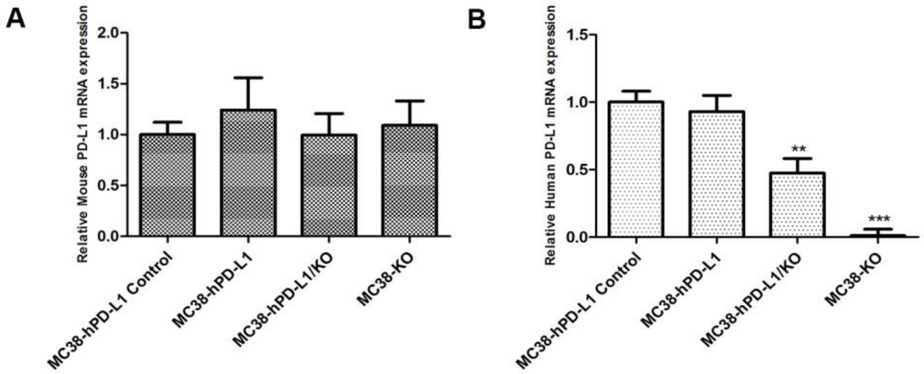

Supplementary Figure 3. RT-PCR quantitative analysis. (**A**) Quantitative analysis of mouse PD-L1 in MC38 tumors. (**B**) Quantitative analysis of human PD-L1 in MC38 tumors. ***P <0.001, **P <0.01, *P <0.05.
